# Supplementary material for: Use of multiple covariates in assessing treatment-effect modifiers: A methodological review of individual participant data meta-analyses
Source: Res Synth Methods. Author manuscript; Available in PMC 2024 Dec 10. (PMC7617190; doi:10.1002/jrsm.1674)
Supplement: Supporting information [file EMS200030-supplement-Supporting_information.docx]

Appendices

# Appendix S1: Data extraction form for IPD meta-analysis studies

## Section A: General information

| **Administrative** | |
| --- | --- |
| **Date extracted** |  |
| **Name of person extracting** |  |
| **First author** |  |
| **Research group / collaboration name** |  |
| **IPD MA characteristics** | |
| **Publication Year** |  |
| **Medical Field** |  |
| **Number of datasets analysed** |  |
| **Number of participants in those datasets** | Min: Max: |
| **Number of participants analysed from those datasets** | Min: Max: Total: |
| **Dataset identification** | Systematic review COCHRANE  Literature search (*includes if an existing IPD dataset was used but updated with a literature search)  Data sharing platform  Collaboration – Prospective IPD with shared protocol  Collaboration – existing IPD dataset (*includes studies using data from a previously published IPD analysis, e.g. done in the same research unit)  Research program  Trial registry database  Patient registry database  Company database  Not reported |
| **PROSPERO ID (if given)** |  |
| **Types of study data included** | RCTs only  Any other study types |
| **IPDMA approach** | 1 stage, fixed effects  1 stage, random effects  2 stage, fixed effects  2 stage, random effects  Trial effect ignored |
| **General modelling approach** |  |
| **Data format of primary outcome** | Binary  Categorical  Continuous  Time to event |
| **Is any outcome reported a composite?** | Yes  No |
| **Sample size considerations** | Power calculation  Post hoc power assessment  Limited to general talk of “IPDs” have larger power compared to aggregate Metanalysis or single trials  None (*includes if discussion mentions that the study may not have had enough power to detect an effect size) |
| **Is multiple testing accounted for?** | Yes  No  Not mentioned |
| **Complex associations** | Non-linear effects – GO TO SECTION B  Effect modification – GO TO SECTION C  Subgroup analysis without comparison test  Neither  *NOTE: effect modification is not considered between treatment effect and trial. This is usually done to test homogeneity and does not involve interest in identification of effect modifiers*. |

## Section B: Complete only for papers assessing non-linear effects

| **Analysis approach for investigation of non-linear trends** |  |
| --- | --- |
| **Justification for analysis method** |  |

## Section C: Complete only for papers assessing effect modification

| **Type of covariate in the effect modification (tick multiple)** | Continuous variable (e.g. age, weight)  Categorised continuous variable (e.g. age <35 vs age 35+)  Categorical variable (e.g. gender, marital status) |
| --- | --- |
| **Number of effect modifications considered in total** |  |
| **Number of outcomes looked at for effect modification** |  |
| **Analysis approach for investigation of effect modification** |  |
| **Are additional covariates included beyond the treatment-covariate interaction for any effect modification?** |  |
| **If so, how are these covariates selected?** | Univariate analysis  Stepwise procedure  Determined a priori  Other, please describe……………………………………………………… |
| **If so, how are these covariates included?** | Additional variables included in the model  3-way interaction (2 covariates interacted with treatment)  Multiple 2-way interactions included in the model  A higher-order factorial covariate constructed from two other categorical covariates  Other, please describe……………………………………………………… |
| **If so, are reasons reported? What are they?** |  |

## Section D: Contact details of IPD meta-analysis team

| **Corresponding authors name, email, and job/study role** |  |
| --- | --- |
| **Principal investigator’s name and email. If corresponding author is principal investigator then leave blank** |  |
| **Lead statistician’s name and email. If corresponding author is lead statistician, then leave blank** |  |

# Appendix S2: Other medical fields (from Table 1)

**Table S1:** Medical fields that occurred in two of less IPD meta-analyses

|  | **Total (n=17)** |
| --- | --- |
| Addiction/Alcoholism | 1 (6%) |
| Complementary and alternative medicine | 2 (12%) |
| Dentistry | 1 (6%) |
| Gastroenterology | 1 (6%) |
| Infection | 1 (6%) |
| Nephrology | 2 (12%) |
| Nutrition | 2 (12%) |
| Respiratory | 2 (12%) |
| Rheumatology | 1 (6%) |
| Sleep Medicine | 1 (6%) |
| Surgery | 2 (12%) |
| Virology | 1 (6%) |

All data are frequency (%)
IPD refers to individual participant data

# Appendix S3: Models used in application to exemplar dataset

**Table S2:** Statistical models fitted on exemplar dataset

| **Model** | **Approach** | **Description** | **Example two-stage linear model** |
| --- | --- | --- | --- |
| 1 | 1 | Single interaction model (unadjusted) | **Stage 1:** $Y_{ij}=\alpha_{i}+ \beta_{1i}{cov1}_{ij}+ \theta_{i}x_{ij}+ \gamma_{icov1}x_{ij}{cov1}_{ij}$  **Stage 2:** Pool $\gamma_{icov1}$ in a meta-analysis to estimate $\boldsymbol{\gamma}_{\boldsymbol{cov}\boldsymbol{1}}$  Here, $cov1$ is our $z$ |
| 2 | 1 | Single interaction model (unadjusted) | **Stage 1:** $Y_{ij}=\alpha_{i}+ \beta_{1i}{cov2}_{ij}+ \theta_{i}x_{ij}+ \gamma_{icov2}x_{ij}{cov2}_{ij}$  **Stage 2:** Pool $\gamma_{icov2}$ in a meta-analysis to estimate $\boldsymbol{\gamma}_{\boldsymbol{cov}\boldsymbol{2}}$  Here, $cov2$ is our $z$ |
| 3 | 2 | Single interaction model (adjusted) | **Stage 1:** $Y_{ij}=\alpha_{i}+ \beta_{1i}{cov1}_{ij}+\beta_{2i}{cov2}_{ij}+\theta_{i}x_{ij}+\gamma_{icov1}x_{ij}{cov1}_{ij}$  **Stage 2:** Pool $\gamma_{icov1}$ in a meta-analysis to estimate $\boldsymbol{\gamma}_{\boldsymbol{cov}\boldsymbol{1}}$  Here, $cov1$ is our $z$; $cov2$ is our $w$ |
| 4 | 2 | Single interaction model (adjusted) | **Stage 1:** $Y_{ij}=\alpha_{i}+ \beta_{1i}{cov2}_{ij}+\beta_{2i}{cov1}_{ij}+\theta_{i}x_{ij}+\gamma_{icov2}x_{ij}{cov2}_{ij}$  **Stage 2:** Pool $\gamma_{icov2}$ in a meta-analysis to estimate $\boldsymbol{\gamma}_{\boldsymbol{cov}\boldsymbol{2}}$  Here, $cov2$ is our $z$; $cov1$ is our $w$ |
| 5 | 3 | Multiple interactions model | **Stage 1:** $Y_{ij}=\alpha_{i}+ \beta_{1i}{cov1}_{ij}+\beta_{2i}{cov2}_{ij}+ \theta_{i}x_{ij}+ \gamma_{icov1}x_{ij}{cov1}_{ij}+\gamma_{icov2}x_{ij}{cov2}_{ij}$  **Stage 2:** Pool $\gamma_{icov1}$ in a meta-analysis to estimate $\boldsymbol{\gamma}_{\boldsymbol{cov}\boldsymbol{1}}$ and Pool $\gamma_{icov2}$ in a meta-analysis to estimate $\boldsymbol{\gamma}_{\boldsymbol{cov}\boldsymbol{2}}$ |

$Y_{ij}$ is the outcome, which is continuous in this exemplar. $\alpha_{i}$ is intercept for each trial. $z$ is the potential effect modifier. $\theta$ is the treatment effect, with $x_{ij}$ an indicator variable for treatment (Yes, No). $w$ is the potential participant-level confounding covariate (or other potential effect modifier). $\gamma$ is the coefficient for the within-trial interaction. $cov1$ refers to Covariate 1; $cov2$ refers to Covariate 2.

The coefficient of interest is bolded for each model (e.g., $\boldsymbol{\gamma}_{\boldsymbol{cov}\boldsymbol{1}}$).
